# Emerging Research in Chronic Pruritus: From Bedside to Bench and Back Again

**DOI:** 10.3390/medicines7050024

**Published:** 2020-04-29

**Authors:** Kyle A. Williams, Shawn G. Kwatra

**Affiliations:** Department of Dermatology, Johns Hopkins University School of Medicine, Baltimore, MD 21287, USA

**Keywords:** pruritus, itch, treatment, therapeutic, pathogenesis, epidemiology

## Abstract

This *Medicines* special issue highlights emerging research spanning from epidemiology to diagnostic workup, pathogenesis, and therapeutics for patients suffering from chronic pruritus. The special issue contains 13 articles reporting relevant epidemiologic and experimental data on chronic pruritus.

## 1. Introduction 

This editorial serves as an introduction to the special issue “Pathogenesis and Treatment of Chronic Pruritus” and contains an overview of various known causes of chronic pruritus and emerging therapeutics. Chronic pruritus is itch that lasts greater than six weeks and is associated with a variety of dermatologic, systemic, neurologic, and psychiatric etiologies. Itch negatively impacts patient quality of life and has devastating psychosocial consequences. The manuscripts published in this special issue are also a showcase of the current understanding of the pathogenesis of chronic pruritus, along with its epidemiology, diagnostic workup, and therapeutic approaches used to treat chronic pruritus.

## 2. Epidemiology

Chronic pruritus can arise in association with many disease processes and affects many patient populations. Thus, epidemiologic studies are needed to gain a better understanding of the burden of disease. In this special issue, a cross-sectional study of over 18,000 itch patients seen at a tertiary care center showed that African American and female patients were more likely to experience pruritus than their white or male counterparts [1]. Furthermore, African American patients were less likely to see a dermatologist than other racial groups, suggesting disparities in care [1]. Additionally, African Americans were more likely to be diagnosed with prurigo nodularis (PN), lichen planus (LP), and atopic dermatitis (AD), which highlight areas for future translational studies. Finally, females were more likely to have comorbid autoimmune or psychiatric conditions [1]. This study’s results were supported when their data was compared to nationally available data.

A major focus of this special issue is placed on prurigo nodularis. PN is a chronic itchy skin condition characterized by severely pruritic nodules that cause a significant negative impact on patient quality of life [2,3]. Kwon et al. performed a systematic review to provide a thorough testing and diagnostic evaluation algorithm for PN patients [4]. In particular, the study highlighted the need for baseline laboratory screening with complete blood cell count (CBC), complete metabolic panel (CMP), thyroid, liver, and kidney function tests, HIV serology, and hepatitis B and C serologies. Additional testing may be indicated based on individual patient’s clinical history and review of systems [4]. 

Whang et al. sought to better characterize the epidemiology and disease burden of this condition by performing a cross-sectional study of the 2016 National Inpatient Sample [5]. Their study found that patients with PN accounted 3.7 inpatient visits per 100,000 discharges nationally [5]. Results also showed that patients diagnosed with PN were more likely to be African American or Asian, have longer and more costly hospital stays and be admitted for HIV complications [5]. These results are in line with prior studies on racial differences in prurigo nodularis [6]. 

Building on these findings, Hughes et al. conducted a cross-sectional analysis on the association of PN with various etiologies of peripheral neuropathy [7], and found significant associations with diabetes mellitus, chronic kidney disease (CKD), human influenza virus, metronidazole use, and hypothyroidism [7]. When compared to AD and psoriasis, the PN cohort was more likely to have chronic kidney disease and HIV [7]. 

Another skin condition featured in this special Issue is mycosis fungoides (MF). Patients with MF experience increased rates of cardiovascular disease and mortality when compared to healthy controls [8,9]. Recent studies have shown a relationship between MF and inflammatory disorders like psoriasis [9,10]. Given the chronic nature of this disease, comorbid conditions can significantly add to patient burden. Therefore, Kaul et al. performed a cross sectional study to evaluate 580 adult patients with diagnosed MF, to identify the common illnesses associated with MF and any racial differences in comorbid disease [11]. This study found that MF was strongly associated with lymphomatoid papulosis, Hodgkin’s disease, congestive heart failure, hypertension, and hyperlipidemia compared with healthy controls [11]. Of note, the association between MF and lymphomatoid papulosis was seen in Caucasian and not African American patients [11]. The study provides valuable epidemiologic information on MF that can be used by clinicians managing this condition [11]. 

Malignancy is also associated with chronic pruritus [12,13]. The association between pruritus and cancer in adults is well recognized in the literature, in particular, with hematological malignancies such as Hodgkin’s lymphoma, leukemia, and cutaneous T-cell lymphoma [13]. However, the prevalence of specific malignancy subtypes differ significantly between the pediatric and adult patient populations. Belzberg et al. performed a retrospective study to assess the association between pruritus and malignancy in pediatric patients [14]. This study found that pediatric patients with pruritus were 13 times more likely to have concomitant malignancy compared to pediatric patients without pruritus [14]. In the pediatric population, the correlation between pruritus and malignancy was strongest for cancers of the bone, skin, liver, and blood, as well as leukemia, non-Hodgkin’s, and Hodgkin’s lymphoma [14]. Pruritus may precede malignancy, concurrently appear, or also arise as a consequence of an adverse reaction of treatment [14]. 

Adverse drug reactions are also a well-recognized etiology of pruritus, and it has been shown by previous studies that pruritus accounts for over 10% of cutaneous drug reactions [15]. These adverse reactions can be chronic or acute and the pruritus generated can occur via direct skin inflammation or through systemic mediators depending on the causative drug [15]. In addition to the significant impact on quality of life pruritus can have, drug-induced pruritus also makes patients less compliant to their medication regimens [16]. Huang et al. performed a study to assess the rates of pruritus associated with commonly prescribed medications [16]. Using retrospective data of 9802 patients with pruritus after drug initiation, they found the highest rates of pruritus in patients taking heparin, trimethoprim-sulfamethoxazole, and calcium channel blockers [16]. They also found that of the pruritic patients a significantly larger proportion of them were female or African American [16]. 

Another class of medication known to be associated with drug induced pruritus are the Epidermal growth factor receptor (EGFR) inhibitors [17]. These medications, such as erlotinib, are often used as chemotherapeutic agents in cancer patients, but a recent survey showed that the cutaneous toxicities of these drug have led to clinicians having to adjust dosages and even discontinue them in patients experiencing these side effects [18]. One class of medications that have been suggested as a therapeutic agent for EGFR-associated pruritus are neurokinin-1 receptor (NK1R) antagonists [19]. Case series studies have reported efficacy in treating chronic itch with NK1R antagonists [20]. Additionally, a 2012 clinical trial supported the efficacy of the NK1R antagonists aprepitant in reducing pruritus caused by EGFR inhibitor therapy [21]. In an effort to better understand the antipruritic effect of aprepitant and its effect on human keratinocytes, Kwatra et al. performed reverse phase protein arrays (RPPA) to analyze its effect on these cells [22]. Results of this study found that aprepitant is a partial agonist of EGFR, and the researchers believe this action is responsible for its antipruritic effects [22]. This supports the need for further research on aprepitant as an itch fighting medication in vivo. 

## 3. Pathophysiology 

The pathophysiology of chronic pruritus is not yet fully understood but involves a complex interplay between the immune and neurologic systems and the skin [1]. As the pathogenesis of pruritus can differ based the etiology, this issue had multiple articles on itch subtypes. A systematic review was done to analyze current literature available on the characteristics and pathophysiological mechanisms of itch in chronic wounds [23]. This article suggested that a number of factors influence itch in chronic wounds including wound area, necrotic tissue amount, exudate amount, peripheral tissue edema, sclerosis, granulation tissue, perilesional skin characteristics, neuropathic changes, and dressing sensitization [23]. They also noted that there are currently no standards for preventing and managing itch in chronic wounds [23]. 

The relationship between nerve fibers and itch has been implicated in several pruritic conditions. Indeed, several itchy conditions have altered neural architecture as well as associated itch, and in some cases also an accompanying burning or pain sensations [24,25]. Chronic kidney disease-associated pruritus CKD-aP is thought to involve an alteration in nerve fibers and increased amounts of circulating pruritogens such as uremic toxins, which also stimulate reactive oxygen species (ROS) [26]. One study sought to further understand how the interact between uremic toxins and nerve fibers in patients with CKD-aP resulted in itch [27]. Momose et al. obtained skin biopsies from the forearm and elbow of two cohorts of patients. One group with CKD-aP and one with CKD patients not experiencing pruritus. After performing quantitative real-time polymerase chain reaction (RT-PCR) on these samples, the researchers found that the Cav3.2 T-type calcium channel, a channel only found in peripheral nerves of the skin that is associated with itch, was significantly higher in patients with CKD-aP than CKD patients without pruritus [27]. The researchers believe uremic toxins act on these ion channels in peripheral nerve endings and may serve as targets for future drugs to fight itch [27]. 

## 4. Treatment

With currently no U.S. Food and Drug Administration (FDA)-approved medications to specifically treat chronic itch as an indication, physicians are forced to use off label therapies [3]. Topical corticosteroid therapy has long been a first line therapy [28]. There are several additional topical agents used off label which Harrison et al. reviewed, including medications such as topical calcineurin inhibitors, capsaicin, ceramide, pine tar-based preparations, and doxepin [28].

The IL-4 receptor is another potential target for therapies aimed at alleviating chronic itch. IL-4 is believed to be a key upstream mediator of chronic pruritus associated with AD and downstream JAK signaling [29]. A case series published in this special issue reports significant itch improvement in a variety of pruritic conditions including, PN, LP, and uremic pruritus with dupilumab therapy [29]. 

Several antidepressants have also shown some benefits in treating itch [30,31,32]. Khanna et al. performed a systematic review to of the current literature to summarize the efficacy of mirtazapine, a tetracyclic antidepressant, in treating chronic itch [33]. The results suggest that mirtazapine may be an especially good option for recalcitrant itch in patients suffering from nocturnal itch who are underweight, as mirtazapine can cause sedation and weight gain. 

In summary, our special issue highlights recent areas of clinical, translational, and basic science itch research. As itch has a significant effect on quality of life, it is our hope that these studies further stimulate research that may help contribute to the development of novel therapies for the treatment of chronic itch. 
